# Sex-Based Differences in Outcomes of Surgical Aortic Valve Replacement: A Meta-Analysis with Reconstructed Time-to-Event Data

**DOI:** 10.1016/j.cjco.2025.11.002

**Published:** 2025-11-12

**Authors:** Leo N. Consoli, Mir W. Majeed, Eren Cetinel, Pawel Lajczak, Ilias G. Koziakas, Tulio Caldonazo

**Affiliations:** a Federal University of Bahia, Salvador, Brazil; b Government Medical College Srinagar, Srinagar, India; c San Raffaele University, Milan, Italy; d Medical University of Silesia, Katowice, Poland; e Cardiac Surgery Department, Onassis Hospital, Athens, Greece; f Department of Cardiothoracic Surgery, Jena University Hospital, Friedrich Schiller University of Jena, Jena, Germany; g Department of Cardiothoracic Surgery, Weill Cornell Medicine, New York, New York, USA

**Keywords:** surgical aortic valve replacement, aortic valve surgery, valve disease, health equity, healthcare disparities

## Abstract

**Background:**

Results in the literature are mixed on how patient sex impacts the outcomes of surgical aortic valve replacement (SAVR), with a high risk of confounding bias due to different risk profiles at presentation. We aimed to assess short- and long-term outcomes of SAVR in male and female patients.

**Methods:**

We searched PubMed, Embase, and the Cochrane Library for eligible confounder-adjusted studies, including those that used propensity-score-matching, multivariable regressions, and inverse probability of treatment weighting. Meta-analysis was performed for short-term (early mortality and procedural complications) and long-term (late mortality, reoperation, and adverse events) endpoints. We compared endpoints, using risk ratio (RR) for short-term endpoints and hazards ratio (HR) for long-term endpoints. We calculated 95% confidence intervals (CIs) for all outcomes. A meta-analysis of Kaplan-Meier-derived individual patient data was done for long-term mortality.

**Results:**

We included 13 studies (n = 159,415). In the short-term, female patients had a higher mortality rate (RR 1.25 [95% CI 1.19, 1.32], *P* < 0.001), more operative deaths (RR 1.33 [95% CI 1.01, 1.75], *P* = 0.04), and required more blood product use (RR 1.36 [95% CI 1.14, 1.63], *P* < 0.001). Male patients had more transient ischemic attacks (RR 0.74 [95% CI 0.56, 0.99], *P* = 0.04) and acute kidney injury (RR 0.73 [95% CI 0.7, 0.77], *P* < 0.001). At long-term follow-up, male patients had a higher mortality rate (HR 0.93 [95% CI 0.88, 0.98], *P* = 0.001), and more reoperations; no significant differences were seen in the rates of late stroke or bleeding.

**Conclusions:**

This meta-analysis found that, compared to male patients, female patients had higher early mortality but lower late mortality following SAVR.

## Central Illustration

**Figure:**
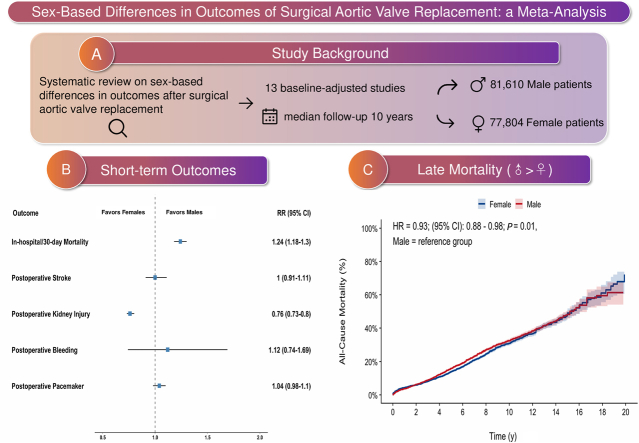

## Résumé

**Contexte:**

Les résultats publiés dans la littérature concernant l'impact du sexe du patient sur les résultats cliniques de chirurgie de remplacement valvulaire aortique sont mitigés, avec un risque élevé de biais de confusion en raison des différents profils de risque à la présentation. Notre objectif était d'évaluer les résultats à court et à long terme de chirurgie de remplacement valvulaire aortique chez les hommes et les femmes.

**Méthodologie:**

Nous avons recherché dans PubMed, Embase et la Cochrane Library des études éligibles ajustées pour les facteurs de confusion, incluant régressions multivariées, appariement par score de propension et pondération par l'inverse de la probabilité d'être traité. Une méta-analyse a été réalisée pour les critères d'évaluation à court terme (mortalité précoce et complications liées à l'intervention) et à long terme (mortalité tardive, réintervention et événements indésirables). Nous avons comparé les critères d'évaluation à l'aide du rapport de risque relatif (RR) pour le court terme et du rapport de risques instantanés (RRI) pour le long terme. Nous avons calculé des intervalles de confiance (IC) à 95 % pour tous les résultats cliniques. Une méta-analyse des données individuelles des patients dérivées des courbes de Kaplan-Meier a été réalisée pour la mortalité à long terme.

**Résultats:**

Nous avons inclus 13 études (n = 159 415). À court terme, les femmes présentaient une mortalité plus élevée (RR 1,25 [IC à 95 % 1,19; 1,32], *p* < 0,001), davantage de décès opératoires (RR 1,33 [IC à 95 % 1,01; 1,75], *p* = 0,04) et ont nécessité un recours plus fréquent aux transfusions sanguines (RR 1,36 [IC à 95 % 1,14; 1,63], *p* < 0,001). Les hommes ont présenté davantage d'accidents ischémiques transitoires (RR 0,74 [IC à 95 % 0,56; 0,99], *p* = 0,04) et d'insuffisance rénale aiguë (RR 0,73 [IC à 95 % 0,7; 0,77], *p* < 0,001). Concernant le suivi à long terme, les hommes présentaient une mortalité plus élevée (RRI 0,93 [IC à 95 % 0,88; 0,98], *p* = 0,001) et davantage de réinterventions ; aucune différence significative n'a été observée pour les taux d'accident vasculaire cérébral tardif ou d'hémorragie.

**Conclusion:**

Cette méta-analyse a révélé que, par rapport aux hommes, les femmes présentaient une mortalité précoce plus élevée, mais une mortalité tardive plus faible après une chirurgie de remplacement valvulaire aortique.

Aortic valve disease is the most common valve pathology in the general population, and its incidence is expected to rise due to aging patterns.^1^ Surgical aortic valve replacement (SAVR) is the standard of care for severe aortic stenosis (AS) or regurgitation; however, despite a similar prevalence of aortic valve disease in the 2 sexes, female patients undergo SAVR less frequently than men and are believed to have worse outcomes,^2^ contributing to both the undertreatment of valve conditions and healthcare disparities.

Although the Society of Thoracic Surgeons (STS) risk score considers female sex a risk factor for early mortality,^3^ studies on SAVR have been inconclusive, with a slight predominance of reports indicating a similar incidence of adjusted in-hospital mortality between sexes.^4^ Additionally, research on long-term, sex-based SAVR outcomes has yielded conflicting results, with some studies reporting comparable survival,^5^ worse survival in female patients,^6^ or even better survival in female patients.^7^ Male and female patients have been observed to differ in age and comorbidity burden at the time of SAVR, which can bias estimates and might be responsible for the heterogeneity in the literature.^2^^,^^4^

To address these gaps in the literature, we conducted a systematic review and meta-analysis of confounder-adjusted studies to evaluate sex-related differences in short- and long-term outcomes following SAVR.

## Methods

This systematic review and meta-analysis was conducted in accordance with the Cochrane Collaboration Handbook for Systematic Reviews of Interventions and adheres to the Preferred Reporting Items for Systematic Reviews and Meta-Analyses (PRISMA) guidelines.^8^^,^^9^ The study protocol was prospectively registered in the International Prospective Register of Systematic Reviews (PROSPERO), under protocol number CRD420250655806. A PRISMA checklist was completed and can be found in Supplemental Appendix S1. As this is a systematic review and meta-analysis of secondary de-identified data, patient consent and institutional review board approval were not necessary.

### Eligibility criteria

We included studies examining sex-based variations in outcomes between male patients (control group) and female patients (intervention/exposure group) treated with SAVR. The study groups were defined exclusively on the basis of biological sex and not patient gender.

The population of interest comprised patients with any aortic valvular disease treated with isolated SAVR or SAVR with concomitant procedures. We included studies published from inception up to March 2025 that reported at least one mortality outcome, categorized into short- and long-term outcomes. To minimize bias due to confounding, we limited inclusion to studies that adjusted for baseline characteristics (eg, propensity-score matching, inverse probability treatment weighting, multivariate regression adjustment). To reduce the risk of publication bias, conference abstracts were also eligible. No language restrictions were applied.

### Search strategy and data extraction

We performed a systematic literature search on PubMed, Embase, and the Cochrane library from inception up to March 2025, using the search strategy detailed in Supplemental Appendix S2. The study selection process was independently performed by 3 reviewers (L.N.C., E.C., and I.G.K.). Disagreements were resolved by consensus. After removing duplicates, the authors screened titles and abstracts and assessed full-text articles for inclusion. We also manually screened the references of included studies for potential records. Data extraction was conducted using a standardized form, by L.N.C. and E.C., revised by M.W.M. We collected information on sample size, study design, patient characteristics, and clinical outcomes. Corresponding authors were contacted by e-mail in case of missing data. The data supporting the findings of this study are available upon reasonable request by e-mail to the first author or the corresponding author.

### Outcomes of interest

We analyzed both short- and long-term outcomes. Short-term outcomes were defined as events occurring during the hospital stay or within 30 days post-procedure. These included early mortality, operative mortality (death occurring during the procedure), stroke, transient ischemic attack, reoperation, major bleeding, blood product use, acute kidney injury, permanent pacemaker insertion, and mean implanted prosthesis size. Long-term outcomes were assessed over a minimum follow-up period of 2 years and included mortality, stroke, reoperation, and major bleeding. Our main outcomes were short- and long-term mortality. Endpoint definitions can be found in Supplemental Table S1.

### Risk-of-bias assessment

Risk-of-bias assessment for the included studies was performed independently by L.N.C. and I.G.K. using the Cochrane tool for assessing **r**isk **o**f **b**ias **i**n **n**onrandomized **s**tudies (ROBINS-I).^10^ Studies were categorized as having a serious, moderate, or low risk of bias based on the following domains: confounding; selection of participants; classification of interventions; deviations from intended interventions; missing data; measurement of outcomes; and selection of the reported result. Any discrepancies were resolved through discussion and re-evaluation.

### Statistical analysis

We used RStudio, version 4.2.2 (R Foundation for Statistical Computing, Vienna, Austria) for all statistical analyses. Initially, we pooled patient and procedural characteristics as proportions with confidence intervals (CIs), if binary, or with means with standard deviations, if continuous. These values were then compared using risk ratio (RR) or mean difference, respectively, in a random-effects model with inverse-variance weighting. To pool treatment effects, we used RR or mean difference for short-term endpoints, and hazard ratios (HRs) for long-term endpoints. For all outcomes, 95% CIs were calculated. We adopted the inverse-variance method, along with a random-effects model, considering a *P*-value < 0.05 to be statistically significant. Statistical heterogeneity among studies was assessed using the Cochran’s Q test, and the Tau^2^, and I^2^ statistics with DerSimonian and Laird’s method. Heterogeneity was classified as not important (I^2^ < 25%), moderate (25% < I^2^ < 50%), substantial (50% < I^2^ < 75%), or considerable (I^2^ > 75%). Publication bias was evaluated using funnel plots and Egger’s regression test. A subgroup analysis was conducted for short-term mortality in isolated SAVR, for patients with AS, and based on outcome definition (in-hospital vs 30 days).

A meta-analysis of Kaplan-Meier-derived individual patient data was performed using the IPDfromKM method.^11^ We extracted data from published curves referrent to a matched population, or adjusted curves generated from multivariate regression. In the latter case, we compared the HR from the extracted data to the one reported in the article’s text, to assess conformity. The proportional hazards assumption was assessed visually and was tested using a Schoenfeld residuals plot and the Grambsch-Therneau test. The HR was obtained via a Cox frailty regression model, with “Study” used as a frailty term (random-effects). The time-dependent HR and the difference in restricted mean survival time were computed using splines from the “survrm” package. Spline models also were used to determine hazard functions for long-term mortality for each study group. A complete description of the statistical methods can be found in Supplemental Appendix S3.

## Results

### Study selection and baseline characteristics

**Figure 1:**
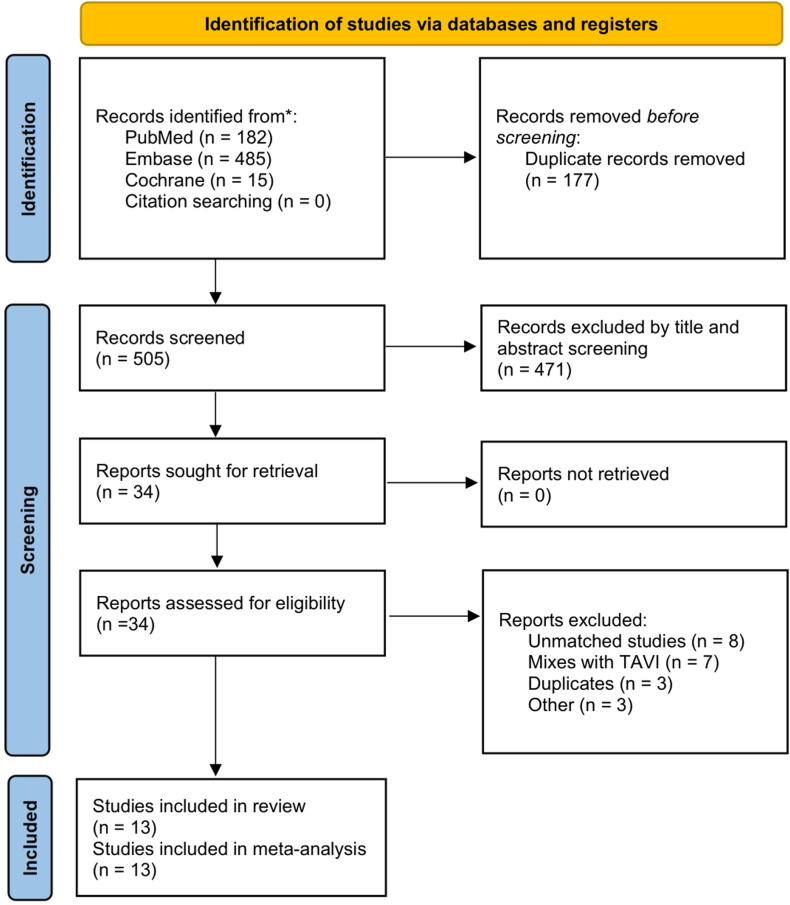
Preferred Reporting Items for Systematic Reviews and Meta-Analyses (PRISMA) flowchart for the systematic review. Sequential steps of study search and triage from databases, with reasons for exclusion. TAVI, transcatheter aortic valve implantation.

The search strategy identified 682 records (Fig. 1). Before screening, 177 duplicate entries were removed. After title and abstract screening, 34 studies underwent full-text review according to the inclusion and exclusion criteria. Of these, 13 studies met all the inclusion criteria.^5^, ^6^, ^7^^,^^12^, ^13^, ^14^, ^15^, ^16^, ^17^, ^18^, ^19^, ^20^, ^21^, ^22^ The analysis included a non-overlapping population of 159,415 patients undergoing SAVR. The characteristics of the included studies are presented in Table 1. Justifications for the exclusion of fully read articles and the references of included studies are in Supplemental Table S2.

**Table 1:** Characteristics of included studies

Study; year	Country; period	Study design	Population	Sample size	Follow-up, y
Andrei et al.^14^ (2015)	USA; 2004–2013	RC—PSM	AS/AR	248	5
Çelik et al.^13^ (2023)	Netherlands; 1987–2017	RC—PSM	AS/AR	1132	20
Chaker et al.^17^ (2017)	USA; 2003–2014	RC—PSM	Severe AS	56,474	DOS
Chang et al.^22^ (2022)	Taiwan; 2000–2013	RC—PSM	AS/AR	4173	DOS
Duncan et al.^20^ (2006)	USA; 1993–2002	RC—PSM	AS/AR	464	DOS
Fialka et al.^16^ (2024)	Canada; 2004–2018	RC—PSM	AS	2544	15
Johnston et al.^19^ (2024)	Sweden; 2008–2016	RC—aMR	AS	11,767	8
Kulik et al.^7^ (2009)	Canada; 1976–2006	PC—aMR	AS/AR	2253	5.6
López-de-Andrés et al.^15^ (2019)	Spain; 2001–2015	RC—PSM	AS	69,294	DOS
Myllykangas et al.^5^ (2020)	Finland; 2004–2014	RC—PSM	AS/AR	5628	15
Pawlik et al.^21^ (2023)	Poland; 2006–2020	RC—PSM	AS/AR	1526	5.8
Zierer et al.^18^ (2024)	Europe/North America; 2019–2021	PC—PSM	AS/AR	676	2
Hernandez-Vaquero et al.^6^ (2021)	Spain; 2000–2018	RC—PSM	AS/AR	3236	15

aMR, adjusted by multivariate regression; AS, aortic stenosis; AR, aortic regurgitation; DOS, duration of stay; PC, prospective cohort; PSM, propensity score-matched; RC, retrospective cohort.

Eleven studies were propensity-score matched, and 2 studies had multivariate adjusted Kaplan-Meier curves. Although most studies had mixed populations, the study by Andrei et al. included only patients with bicuspid aortic valves, and the study by Chaker et al. included patients with severe AS as their population.^14^^,^^17^ The proportion of mechanical to bioprosthetic aortic valves varied considerably across studies. The size of included studies had a large range (248 up to 69,294), with some being single-centre cohorts and others nationwide patient analyses. Nine studies reported Kaplan-Meier data on long-term survival, eight on early mortality, and only 4 studies on short- and long-term adverse events. Table 2 presents pooled patient and procedural characteristics. Study groups were well balanced for most variables, but female patients were slightly older (mean difference of 0.97 years, *P* = 0.015). Time under cardiopulmonary bypass and aortic cross-clamp were shorter in female patients. Characteristics of patients in each study are presented in Supplemental Table S3.

**Table 2:** Pooled patient characteristics

Variables	Overall (N = 159,414)	Male patients (n = 81,610)	Female patients (n = 77,804)	Effect size measure (MD or RR)	*P*
Age, y	68.71 (10.57)	67.96 (10.79)	69.49 (10.27)	MD = 0.97 (0.19–1.76)	**0.015**
LVEF, %	59.75 (10.08)	59.81 (10.34)	59.69 (9.81)	MD = -0.39 (-0.90–0.12)	0.134
Hypertension, %	58.87 (58.55–59.18)	57.60 (57.11–58.09)	60.22 (59.72–60.73)	RR = 1.04 (1.00–1.08)	0.067
Diabetes mellitus, %	23.32 (23.13–23.51)	22.99 (22.69–23.29)	23.67 (23.26–23.98)	RR = 1.00 (0.96–1.05)	0.969
AF, %	34.55 (34.33–34.78)	33.64 (33.29–33.98)	35.53 (35.17–35.89)	RR = 1.02 (0.97–1.07)	0.498
COPD, %	14.80 (14.61–14.99)	14.79 (14.49–15.10)	14.82 (14.51–15.13)	RR = 0.99 (0.96–1.02)	0.605
CAD, %	26.85 (26.60–27.10)	28.52 (28.17–28.87)	25.05 (24.72–25.39)	RR = 0.98 (0.85–1.13)	0.820
BMI, kg/m^2^	28.77 (5.39)	28.64 (4.89)	28.91 (5.90)	MD = 0.11 (-0.11–0.32)	0.326
Aortic regurgitation, %	1.21 (0.24–2.18)	1.23 (0.10–12.99)	1.19 (0.11–11.81)	RR = 0.88 (0.73–1.06)	0.185
Mechanical valve, %	43.14 (42.92–43.37)	42.81 (42.45–43.17)	43.50 (43.14–43.87)	RR = 0.98 (0.93–1.02)	0.315
CPB time, min	119.39 (46.33)	122.95 (46.6)	115.69 (46.01)	MD = -6.48 (-10.05– -2.91)	**< 0.001**
Clamp time, min	83.81 (31.59)	85.89 (30.97)	81.56 (32.12)	MD = -4.77 (-7.03 – -2.52)	**< 0.001**

Data are presented as means with standard deviations or with 95% confidence intervals. Boldface indicates significance.

AF, atrial fibrillation; BMI, body mass index; CAD, coronary artery disease; COPD, chronic obstructive pulmonary disease; CPB, cardiopulmonary bypass; LVEF, left ventricle ejection fraction; MD, mean difference; RR, risk ratio.

### Short-term endpoints

**Figure 2:**
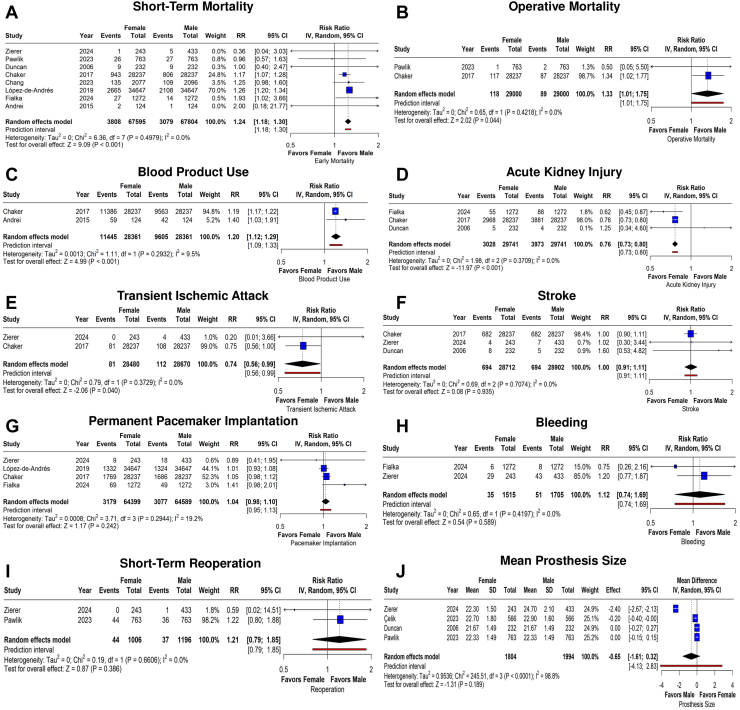
Forest plots comparing the risk of short-term outcomes between male and female patients: (**A**) mortality; (**B**) operative mortality; (**C**) blood product use; (**D**) acute kidney injury; (**E**) transient ischemic attack; (**F**) stroke; (**G**) permanent pacemaker implantation; (**H**) bleeding; and (**I**) short-term reoperation. (**J**) Forest plot comparing the difference in mean prosthesis size. CI, confidence interval; IV, inverse variance; RR, risk ratio; SD, standard deviation.

The pooled analysis showed that female patients had significantly higher risks for early mortality (RR 1.25; 95% CI 1.17-1.32; *P* < 0.001; I^2^ = 0%; Fig. 2A) and operative death (RR 1.33; 95% CI 1.01-1.75; *P* = 0.044; I^2^ = 0%; Fig. 2B). Additionally, females required significantly more blood product transfusions (RR 1.36; 95% CI 1.14-1.63; *P* < 0.001; I^2^ = 21.1%; Fig. 2C). In the subgroup of patients undergoing isolated SAVR, a higher mortality risk was maintained in female patients (RR 1.18; 95% CI 1.08-1.28; *P* < 0.001; I^2^ = 0%; Supplemental Fig. S1). For patients with AS, the mortality results were similar to the overall study findings. However, mixed patients with stenosis and regurgitation had no significant difference in mortality. Most studies reported outcomes during the hospital stay, and these early mortality findings matched the overall analysis. Studies that evaluated 30-day mortality, however, found no sex-based difference (Supplemental Fig. S2).

In contrast, male patients experienced higher rates of acute kidney injury (RR 0.73; 95% CI 0.70-0.77; *P* < 0.001; I^2^ = 0%; Fig. 2D) and transient ischemic attacks (RR 0.74; 95% CI 0.56-0.99; *P* = 0.04; I^2^ = 0%; Fig. 2E). The risks of stroke (RR 1.0; 95% CI 0.9-1.12; *P* = 0.935; I^2^ = 0%; Fig. 2F), permanent pacemaker implantation (RR 1.04; 95% CI 0.97-1.11; *P* = 0.244; I^2^ = 19.8%; Fig. 2G), major bleeding (RR 1.12; 95% CI 0.71-1.77; *P* = 0.614; I^2^ = 0%; Fig. 2H), and early reoperation (RR 1.21; 95% CI 0.79-1.85; *P* = 0.386; I^2^ = 0%; Fig. 2I) were similar between sexes. Mean implanted prosthesis size was similar in the groups (mean difference, –0.65 mm; 95% CI –1.61-0.32; *P* = 0.189; I^2^ = 98%; Fig. 2J). Table 3 summarizes the short-term outcomes of individual studies. Heterogeneity was generally not important or was moderate (I^2^ < 25% for most outcomes), supporting the robustness of our findings, with the exception of prosthesis size, which had considerable heterogeneity. In leave-one-out analysis, all outcomes remained stable independent of the omitted study (Supplemental Figs. S3-S7), but some endpoints could not be analyzed as they were reported in only 2 studies.

**Table 3:** Postoperative outcomes in individual studies

Study	Mortality	Operative mortality	Bleeding	Blood product	Stroke	TIA	AKI	Reoperation	PPM	Prosthesis size, mm
Andrei et al.^14^ (2015)	1.61/0.80	NR	NR	47.58/33.87	NR	NR	NR	NR	NR	NR
Chaker et al.^17^ (2017)	3.34/2.85	0.41/0.30	NR	40.32/33.86	2.41/2.41	0.28/0.38	10.51/13.74	NR	6.26/5.97	NR
Zierer et al.^18^ (2024)	0.41/1.15	NR	11.9/9.93	NR	1.64/1.61	0/0.92	NR	0/0.23	3.70/4.15	22.30/24.70
Chang et al.^22^ (2022)	6.49/5.20	NR	NR	NR	NR	NR	NR	NR	NR	NR
Duncan et al.^20^ (2006)	3.88/3.88	NR	NR	NR	3.44/2.15	NR	2.15/1.72	NR	NR	21.67/21.67
Fialka et al.^16^ (2024)	2.12/1.10	NR	0.47/0.62	NR	NR	NR	4.32/6.91	NR	5.42/3.85	NR
López-de-Andrés et al.^15^ (2019)	7.69/6.08	NR	NR	NR	NR	NR	NR	NR	3.84/3.82	NR
Pawlik et al.^21^ (2023)	3.40/3.53	0.13/0.26	NR	NR	NR	NR	NR	5.76/4.71	NR	22.33/22.33
Çelik et al.^13^ (2023)	NR	NR	NR	NR	NR	NR	NR	NR	NR	22.70/22.90

Values are %, unless otherwise indicated, and are for female patients/male patients.

AKI, acute kidney injury; NR, not reported; PPM, permanent pacemaker implantation; TIA, transient ischemic attack.

### Long-term endpoints

**Figure 3:**
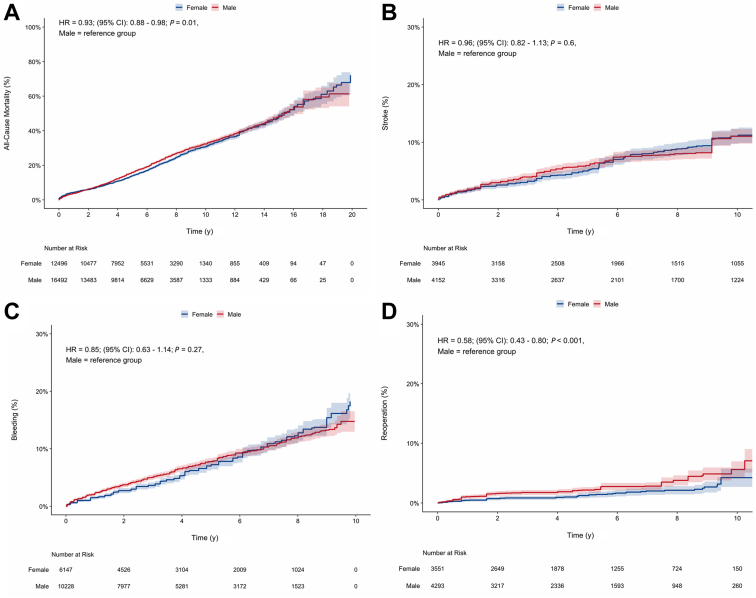
Kaplan-Meier curves for the cumulative incidence of events in male and female patients, for the following outcomes: (**A**) all-cause mortality, showing a lower hazard in female patients; (**B**) stroke; (**C**) major bleeding; and (**D**) reoperation, showing a lower hazard in female patients. CI, confidence interval; HR, hazards ratio.

**Figure 4:**
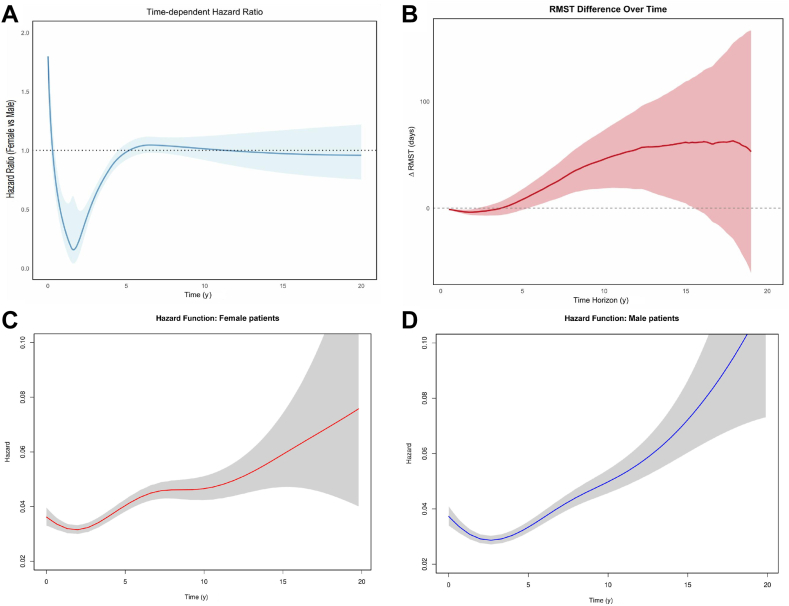
Survival analysis. (**A**) Time-dependent hazard ratio function. (**B**) Time-dependent difference in restricted mean survival time (RMST). (**C**) Hazard function in female patients. (**D**) Hazard function in male patients.

The reconstructed Kaplan-Meier meta-analysis included 28,970 patients (16,483 male patients, 12,487 female patients), with 162,234 patient-years. Over 20 years of follow-up, men exhibited a higher mortality hazard (HR 0.93; 95% CI 0.88-0.98; *P* < 0.01; Fig. 3A). When analyzed over time, the HR was initially much higher in female patients, and then became lower at approximately 2 years, and then became neutral at around 5 years (Fig. 4A). A positive difference in restricted mean survival time was observed until 15years of follow-up care, but the overall result was not statistically significant (0.11 years; 95% CI: –0.22-0.45; *P* = 0.50; Fig. 4B). The hazard functions show higher initial hazards in female patients, which become comparable with those of male patients, and ultimately lower at late follow-up evaluation (Fig. 4, C and D). A landmark analysis with time truncated at 14 years indicated that no survival benefit occurred after this time point (Supplemental Fig. S8).

The incidence of late stroke (HR 1.22; 95% CI 0.98-1.52; *P* = 0.08; Fig. 3B) and major bleeding (HR 0.85; 95% CI 0.63-1.14; *P* = 0.27; Fig. 3C) was comparable between groups, whereas reoperations were less frequent in female patients (HR 0.58; 95% CI 0.43-0.80: *P* < 0.001; Fig. 3D).

### Risk of bias

**Figure 5:**
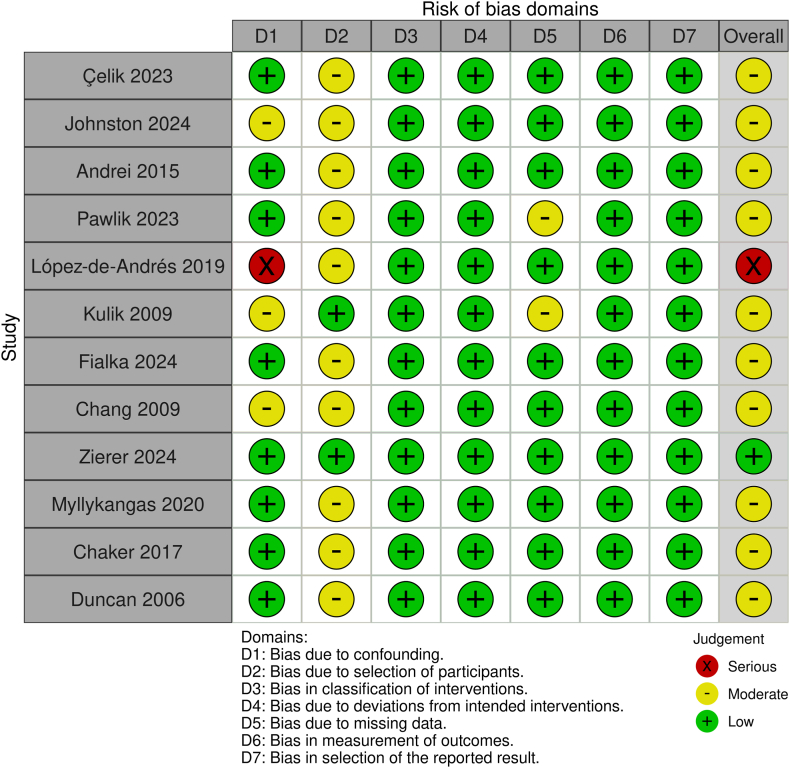
“Traffic light” plot. Risk-of-bias analysis of included studies using the Cochrane tool for assessing **r**isk **o**f **b**ias **i**n **n**onrandomized **s**tudies (ROBINS-I).

The included studies were assessed for risk of bias using the ROBINS-I tool, following Cochrane's guidelines (Fig. 5). One study presented serious concerns, largely due to confounding bias, and one study was at low risk of bias. The remaining studies were judged to be at moderate risk of bias due to confounding, selection bias, or bias due to missing data.

In the funnel plot for short-term mortality, studies were distributed symmetrically according to effect sizes and standard errors, suggesting a low risk of publication bias (Supplemental Fig. S9; Egger’s test *P*-value = 0.76). The distribution was also symmetrical in the funnel plot for long-term mortality, with a low risk of publication bias (Supplemental Fig. S10; Egger’s test *P*-value = 0.82). We performed a sensitivity analysis by excluding the studies rated as being at serious risk of bias, which contributed to the early mortality and permanent pacemaker outcomes, and found results similar to those of the overall analysis (Supplemental Fig. S11).

### Causal analysis

We constructed a directed acyclic graph to identify potential confounders, mediators, and colliders in the relationship between sex (exposure) and postoperative outcomes after SAVR (Supplemental Fig. S12). The graph highlights age, comorbidities, socioeconomic status, and surgeon and/or hospital factors as the main potential confounders. The graph also shows that prosthesis size, concomitant procedures, and periprocedural care are probable mediators on the causal path from sex to outcomes, and that selection into SAVR (referral and surgical selection) is a collider that can bias results. Most included studies adjusted for age and comorbidities (Supplemental Table S4). However, 2 studies adjusted for prosthesis size, and 3 adjusted for concomitant coronary bypass surgery, indicating that these data might be estimating a direct effect of sex, rather than the total effect. We performed a subgroup analysis including only studies that estimate a total effect (Supplemental Fig. S13) and found results similar to those of the main analysis. The major highlights of this work are summarized in the Central Illustration.

**Central Illustration:**
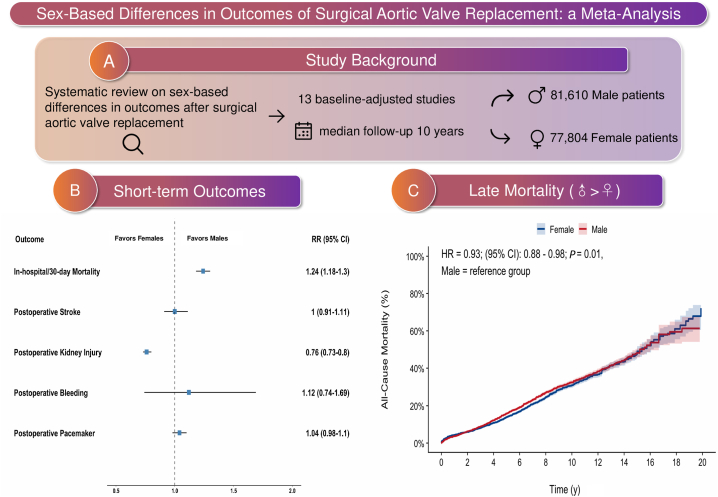
Summary of the research question, methodology, and main findings. (**A**) Background of the study, with the question, methods, and results. (**B**) Short-term endpoints (in-hospital or at 30 days). (**C**) Kaplan-Meier curve for long-term mortality comparing male patients (**blue line**) and female patients (**red line**). CI, confidence interval; HR, hazards ratio; RR, risk ratio.

## Discussion

In this meta-analysis of 13 studies including 159,415 patients, we found the following. In the short term, female patients had significantly higher rates of early mortality, and operative death, and a greater need for blood products, whereas male patients had higher rates of transient ischemic attacks and acute kidney injury. Stroke, pacemaker insertion, bleeding, and reoperation rates were similar between the groups. In the long-term follow-up, men had a significantly higher hazard of death and reoperation, whereas no significant differences were observed in rates of stroke and bleeding.

The previous meta-analysis on this topic pooled 7 studies and found worse late survival in female patients, but no differences in early mortality.^12^ This discrepancy, compared to our results, may be explained by the larger number of studies included in our analysis and the increased sample size, which improved the detection of early outcome event rates. Additionally, the inclusion of only confounder-adjusted studies may account for the difference in late-mortality findings.

Female sex is considered a risk factor for in-hospital mortality in the Society of Thoracic Surgeons risk model, based primarily on registry data from the Adult Cardiac Surgery Database. We found that female patients had higher early mortality after SAVR, both in isolated procedures and when combined with concomitant surgeries, even after baseline matching. This finding suggests that worse outcomes in female patients are due not solely to a higher risk profile at presentation but also to sex-specific differences in how individual risk factors influence outcomes.^4^ Notably, referral bias often leads female patients to undergo SAVR at more advanced stages of valve disease.^2^ Although the subgroups of studies that mixed AS and regurgitation, or of those reporting 30-day mortality, found no sex-based difference, this finding might be attributed to the smaller sample size of these subgroups.

Anatomic differences may further contribute to higher surgical risk in female patients. Smaller body size affects the aortic annulus,^23^ making procedures more technically challenging and increasing the likelihood of receiving smaller prosthetic valves,^24^ which is a known risk factor for mortality. A “challenge-skill balance” issue is plausible in this situation.^25^ In large database analyses, such as those included in this review, average-skilled surgical teams might be faced with the above-average challenge of operating on female patients, resulting in below-average outcomes. A point to note is that, despite the matching performed in these studies, matching male and female patients by body size is typically not possible, due to insufficient overlap. Additionally, sex-related differences in cardiopulmonary bypass management, cardioplegia strategies, and postoperative care may impact outcomes. For instance, Chaker et al. reported longer intensive care unit stays in female patients following isolated SAVR.^17^

In male patients, the higher rates of acute kidney injury and ischemic events may be linked to a greater burden of atherosclerosis. Male patients also are more likely to have undergone prior percutaneous coronary intervention and to require concomitant coronary bypass during SAVR.^17^

Previous research has shown mixed results regarding the long-term outcomes of SAVR, with most studies reporting similar survival rates between male and female patients. However, considering the differences in life expectancy between the sexes, this apparent neutrality in outcomes might actually reflect a worse relative result in female patients.^26^ In this context, the higher hazard of death in male patients could indicate true parity in survival following SAVR. Sex differences in the pathophysiology of AS also should be considered. Female patients tend to experience slower hemodynamic progression, better left ventricular ejection fraction, improved ventricular remodelling, and less fibrosis than male patients,^24^ which may contribute to better cardiac recovery following SAVR. The reason the survival advantage was not sustained after 14 years was not clear, but a potentially higher incidence of prosthesis-patient mismatch in female patients, different comorbidity profiles, and variations in secondary operations might explain the divergence in findings. Additionally, the survival advantage might have been driven by the use of bioprosthetic valves in female patients,^7^^,^^19^ who are typically elderly, so this time period might have overlapped with the natural life expectancy of these patients. Therefore, most patients after 14 years of follow-up could have been those who received mechanical valves and did not differ in mortality rate.

Among the included studies, the major outlier was by Hernandez-Vaquero et al.^6^ They analyzed data from 3236 patients aged 50-65 years enrolled in a retrospective registry and reported worse long-term survival in female patients. Although male patients had a higher comorbidity burden before baseline matching, the authors did not investigate potential reasons for their findings.

A strong but nonsignificant trend was observed for higher rates of late stroke in female patients. Some studies suggest that female patients are at greater risk for neurologic events following cardiac procedures, but our findings did not confirm this.^7^ This discrepancy is likely due to the inclusion of studies from a diverse range of countries with varying referral patterns.

To address this discrepancy, simply reducing SAVR mortality is not sufficient, as worse outcomes for female patients have persisted despite decreasing in-hospital mortality over time.^15^ To improve health equity, future research should focus on understanding how risk factors behave differently based on sex. The use of larger valves and strategies to reduce patient-prosthesis mismatch should be encouraged in female patients, such as pre-procedural computed tomography planning. Efforts also could be made to change referral patterns and promote earlier intervention before the advanced progression of aortic valve disease.

### Limitations

This study has limitations to consider. As a meta-analysis of observational studies, this study carries a risk of confounding and selection bias, despite statistical adjustments and baseline matching. Although a meta-analysis of randomized trials is the gold standard, sex is not a randomizable variable, making such trials unfeasible. We did not have access to patient-level data and instead relied on Kaplan-Meier-derived individual patient data, limiting our ability to perform subgroup analyses. In particular, a subgroup based on prosthesis type is relevant due to the potential for the survival benefit to be driven by the use of biological valves in female patients. Only 2 included studies reported a subgroup analysis based on type of prosthesis used. Additionally, we included large studies with extensive follow-up periods, placing our study at risk of age-period-cohort effects. We also acknowledge that all included studies were conducted in developed countries in North America, Europe, or Asia, where modern, high-performance prostheses are more readily available. As a result, our findings may not be completely generalizable to developing countries. We did not have sufficient data on prosthesis-patient mismatch, paravalvular leak, prosthesis gradient, or structural deterioration over time. Some outcomes were reported by only a small number of studies, and the estimates are driven by the larger study by Chaker et al., so these findings should be taken as, at most, hypothesis-generating. Lastly, significant variation was present in the populations of included studies regarding age, the proportion of AS to aortic regurgitation, and the use of mechanical vs bioprosthetic valves. However, despite these variances, no important heterogeneity was observed for most outcomes.

## Conclusion

In this meta-analysis, we assessed the impact of sex on SAVR outcomes. We included 13 studies with 159,415 patients and found that compared to male patients, female patients had higher early mortality and lower late mortality. Operatory death and blood product use were also higher in female patients, whereas men had more acute kidney injury, transient ischemic attacks, and late reoperations. However, these outcomes are reported in a small number of studies and should be taken as hypothesis-generating. No difference was seen in other endpoints.

## Footnotes

To access the supplementary material accompanying this article, visit *CJC Open* at https://www.cjcopen.ca/ and at https://doi.org/10.1016/j.cjco.2025.11.002.
