# Supplementary material for: Sex-Based Differences in Outcomes of Surgical Aortic Valve Replacement: A Meta-Analysis with Reconstructed Time-to-Event Data
Source: CJC Open. 2025 Nov 12;8(3):344–55. doi: 10.1016/j.cjco.2025.11.002 (PMC12983281; doi:10.1016/j.cjco.2025.11.002)
Supplement: Supplemental Appendix [file mmc1.docx]

**Supplemental Material**

**Table of contents**

**Supplemental Appendix S1: PRISMA checklist .....................................................................2 Supplemental Appendix S2: Search strategy ……………......................................................4 Supplemental Appendix S3: Statistical methods…………………………………………….5 Supplemental Table S1 : Outcome definitions…………………………………………….…6 Supplemental Table S2: Selection justification…………...……........................................….7 Supplemental Table S3: Additional patient data..….…..……...……………………….……8 Supplemental Table S4: Adjustments by study…………...………....................................…9 Supplemental Figure S1……………...….……………………...……………………….……10 Supplemental Figure S2………...….……………………...……………………….……...….10 Supplemental Figure S3………...….……………………...…………………………....…….11 Supplemental Figure S4………...….……………………...…………………………....…….11 Supplemental Figure S5………...….……………………...………………………..………...11 Supplemental Figure S6………...….……………………...………………………..………...12 Supplemental Figure S7………...….……………………...………………….………..……..12 Supplemental Figure S8………...….……………………...………………….………..……..13 Supplemental Figure S9………...….……………………...……………….…………..……..13 Supplemental Figure S10………...….……………………...……………….…………...…...14 Supplemental Figure S11………...….……………………...……………….……….…..…...14 Supplemental Figure S12………...….……………………...………………….………...…...15 Supplemental Figure S13...………………………………...……………………….………...16**

| **Section and**  **Supplemental Appendix S1 - PRISMA Checklist**  **Topic** | **Item #** | **Checklist item** | **Location where item is reported** |
| --- | --- | --- | --- |
| **TITLE** | | |  |
| Title | 1 | Identify the report as a systematic review. | Page 1 |
| **ABSTRACT** | | |  |
| Abstract | 2 | See the PRISMA 2020 for Abstracts checklist. | Page 2 |
| **INTRODUCTION** | | |  |
| Rationale | 3 | Describe the rationale for the review in the context of existing knowledge. | Page 4 |
| Objectives | 4 | Provide an explicit statement of the objective(s) or question(s) the review addresses. | Page 4 |
| **METHODS** | | |  |
| Eligibility criteria | 5 | Specify the inclusion and exclusion criteria for the review and how studies were grouped for the syntheses. | Page 4 |
| Information sources | 6 | Specify all databases, registers, websites, organisations, reference lists and other sources searched or consulted to identify studies. Specify the date when each source was last searched or consulted. | Page 4 |
| Search strategy | 7 | Present the full search strategies for all databases, registers and websites, including any filters and limits used. | Page 5 |
| Selection process | 8 | Specify the methods used to decide whether a study met the inclusion criteria of the review, including how many reviewers screened each record and each report retrieved, whether they worked independently, and if applicable, details of automation tools used in the process. | Page 5 |
| Data collection process | 9 | Specify the methods used to collect data from reports, including how many reviewers collected data from each report, whether they worked independently, any processes for obtaining or confirming data from study investigators, and if applicable, details of automation tools used in the process. | Page 5 |
| Data items | 10a | List and define all outcomes for which data were sought. Specify whether all results that were compatible with each outcome domain in each study were sought (e.g. for all measures, time points, analyses), and if not, the methods used to decide which results to collect. | Page 5 |
|  | 10b | List and define all other variables for which data were sought (e.g. participant and intervention characteristics, funding sources). Describe any assumptions made about any missing or unclear information. | Page 5 |
| Study risk of bias assessment | 11 | Specify the methods used to assess risk of bias in the included studies, including details of the tool(s) used, how many reviewers assessed each study and whether they worked independently, and if applicable, details of automation tools used in the process. | Page 5 |
| Effect measures | 12 | Specify for each outcome the effect measure(s) (e.g. risk ratio, mean difference) used in the synthesis or presentation of results. | Page 5 |
| Synthesis methods | 13a | Describe the processes used to decide which studies were eligible for each synthesis (e.g. tabulating the study intervention characteristics and comparing against the planned groups for each synthesis (item #5)). | Page 6 |
|  | 13b | Describe any methods required to prepare the data for presentation or synthesis, such as handling of missing summary statistics, or data conversions. | Page 6 |
|  | 13c | Describe any methods used to tabulate or visually display results of individual studies and syntheses. | Page 6 |
|  | 13d | Describe any methods used to synthesize results and provide a rationale for the choice(s). If meta-analysis was performed, describe the model(s), method(s) to identify the presence and extent of statistical heterogeneity, and software package(s) used. | Page 6 |
|  | 13e | Describe any methods used to explore possible causes of heterogeneity among study results (e.g. subgroup analysis, meta-regression). | Page 6 |
|  | 13f | Describe any sensitivity analyses conducted to assess robustness of the synthesized results. | Page 6 |
| Reporting bias assessment | 14 | Describe any methods used to assess risk of bias due to missing results in a synthesis (arising from reporting biases). | Page 6 |
| Certainty assessment | 15 | Describe any methods used to assess certainty (or confidence) in the body of evidence for an outcome. | N/A |
| **RESULTS** | | |  |
| Study selection | 16a | Describe the results of the search and selection process, from the number of records identified in the search to the number of studies included in the review, ideally using a flow diagram. | Page 7 |
|  | 16b | Cite studies that might appear to meet the inclusion criteria, but which were excluded, and explain why they were excluded. | Page 7 |
| Study characteristics | 17 | Cite each included study and present its characteristics. | Page 7 |
| Risk of bias in studies | 18 | Present assessments of risk of bias for each included study. | Page 9 |
| Results of individual studies | 19 | For all outcomes, present, for each study: (a) summary statistics for each group (where appropriate) and (b) an effect estimate and its precision (e.g. confidence/credible interval), ideally using structured tables or plots. | N/A |
| Results of syntheses | 20a | For each synthesis, briefly summarise the characteristics and risk of bias among contributing studies. | Pages 8 |
|  | 20b | Present results of all statistical syntheses conducted. If meta-analysis was done, present for each the summary estimate and its precision (e.g. confidence/credible interval) and measures of statistical heterogeneity. If comparing groups, describe the direction of the effect. | Pages 8 |
|  | 20c | Present results of all investigations of possible causes of heterogeneity among study results. | Pages 8 |
|  | 20d | Present results of all sensitivity analyses conducted to assess the robustness of the synthesized results. | Pages 8 |
| Reporting biases | 21 | Present assessments of risk of bias due to missing results (arising from reporting biases) for each synthesis assessed. | Pages 9 |
| Certainty of evidence | 22 | Present assessments of certainty (or confidence) in the body of evidence for each outcome assessed. | N/A |
| **DISCUSSION** | | |  |
| Discussion | 23a | Provide a general interpretation of the results in the context of other evidence. | Page 9-10 |
|  | 23b | Discuss any limitations of the evidence included in the review. | Page 12 |
|  | 23c | Discuss any limitations of the review processes used. | Page 12 |
|  | 23d | Discuss implications of the results for practice, policy, and future research. | Page 10-12 |
| **OTHER INFORMATION** | | |  |
| Registration and protocol | 24a | Provide registration information for the review, including register name and registration number, or state that the review was not registered. | Page 5 |
|  | 24b | Indicate where the review protocol can be accessed, or state that a protocol was not prepared. | Page 5 |
|  | 24c | Describe and explain any amendments to information provided at registration or in the protocol. | N/A |
| Support | 25 | Describe sources of financial or non-financial support for the review, and the role of the funders or sponsors in the review. | Page 13 |
| Competing interests | 26 | Declare any competing interests of review authors. | Page 13 |
| Availability of data, code and other materials | 27 | Report which of the following are publicly available and where they can be found: template data collection forms; data extracted from included studies; data used for all analyses; analytic code; any other materials used in the review. | Page 5 |

*From:*  Page MJ, McKenzie JE, Bossuyt PM, Boutron I, Hoffmann TC, Mulrow CD, et al. The PRISMA 2020 statement: an updated guideline for reporting systematic reviews. BMJ 2021;372:n71. doi: 10.1136/bmj.n71. This work is licensed under CC BY 4.0. To view a copy of this license, visit <https://creativecommons.org/licenses/by/4.0/>

**Supplemental Appendix S2: Search Strategy**

Available below is the search strategy we created for PubMed and modified for the other databases as was necessary using relevant terms and syntax. All references from selected studies were also manually retrieved for “backwards snowballing”.

**PubMed search strategy:** ("aortic stenosis" OR aortic valve disease) AND ("surgical aortic valve replacement" OR SAVR OR AVR OR aortic valve surgery) AND ("sex differences" OR "gender disparities" OR "gender differences" OR "sex-specific outcomes" OR "sex-related differences")

**Embase search strategy:** ('aortic stenosis' OR 'aortic valve disease') AND ('surgical aortic valve replacement' OR 'savr' OR 'avr' OR 'aortic valve surgery') AND ('sex differences' OR 'gender disparities' OR 'gender differences' OR 'sex-specific outcomes' OR 'sex-related differences')

**Cochrane search strategy:** (aortic stenosis OR aortic valve disease) AND (surgical aortic valve replacement OR SAVR OR AVR OR aortic valve surgery) AND (sex differences OR gender disparities OR gender differences OR sex-specific outcomes OR sex-related differences)

**Applied filters:** None.

**Supplemental Appendix S3: Statistical Methods**

**Pairwise meta-analysis:** We pooled patient and procedural characteristics as proportions with confidence intervals (CI), if binary, or means with standard deviations, if continuous. These were then compared using risk ratio (RR) or mean difference (MD), respectively, in a random-effects model with inverse-variance weighting. Following this, we collected the number of events and total number of patients in the exposure (female) and control (male) groups for the short-term outcomes, as well as the mean and standard deviation, if continuous. This data was stored into a prespecified Excel spreadsheet and imported into R studio version 4.2.2. Using the “meta” package, we pooled RR or MD with 95% CIs. The inverse-varience method and random-effects model was applied to all outcomes. Heterogeneity estimators were determined using Der Simonian-Laird’s approach. A funnel plot was generate for the mortality outcomes using the “funnel” function and asymmetry was investigated using Egger’s regression test. To understand how heterogeneity impacted the distribution of effect size estimates, a prediction interval was also calculate for all endpoints. Robustness was explored by way of a leave-one-out analysis and mixed-effects meta-regression. A subgroup analysis for early-mortality was performed by only including cohorts where patients underwent isolated SAVR; studies that adjusted by propensity score-matching versus multivariate regression; and studies that reported in-hospital vs 30-day mortality.

**Reconstructing individual patient data:** For long-term outcomes, we collected the hazards ratios (HR), the lower bound of the confidence interval and the upper bound of the confidence interval. In studies that separated cohorts based on the type of valve received, hazards ratios were extracted and later pooled separately. Kaplan-Meier curves were digitized with the ShinyApp web software and individual-patient-data was reconstructed using the IPDfromKM method. This data was combined into a csv file and imported into R studio.

**Reconstructed IPD meta-analysis:** Initially, we tested the proportional hazards assumption with the “cox.zph” function by visual assessment of Schoenfeld residuals plots. A frailty hierarchical Cox regression model was applied to obtain HRs with the “coxph” function. “Sex” was used as a fixed term and “study” as the frailty term (random-effects). Reconstructed HRs from each individual study were compared with published data for consistency. A difference of 0.02 between reconstructed and study-reported HR was considered acceptable. Splines were used to assess time-dependent variation in the HR and in the difference of restricted mean survival time. Hazard functions were estimated for mortality in men and women. Patient years were calculated by adding up the follow-up times of all patients in the IPD meta-analysis. We used the “survival”, “ggsurvfit”, “survRM2” and “rms” packages.

**Outcome definitions**

**Supplemental Table S1**: Outcome definitions

| **Outcome** | **Definition** |
| --- | --- |
| **Early mortality** | Any in-hospital death or up to 30 days after surgery |
| **Operative death** | Any death in the operating room. |
| **Stroke** | Any stroke diagnosed by an imaging method during hospital stay or up to 30 days after surgery. |
| **Transient ischemic attack** | Any transient cerebrovascular event during hospital stay or up to 30 days after surgery. |
| **Reoperation** | Any reoperation during hospital stay or up to 30 days after surgery. |
| **Major bleeding** | Any clinically significant bleeding (requiring transfusion, reoperation or causing symptoms) during hospital stay, operation or up to 30 days after surgery. |
| **Blood product use** | Any blood product use (plasma, platelets, etc) during hospital stay, operation or up to 30 days after surgery. |
| **Acute kidney injury** | New-onset renal failure (KDIGO criteria) during hospital stay or up to 30 days after surgery. |
| **Permanent Pacemaker** | Implantation of a new pacemaker during hospital stay or up to 30 days after surgery. |
| **Prosthesis size** | Mean post-operative valve prosthetic size |
| **Late mortality** | All-cause mortality at least 1 year after surgery. |
| **Late stroke** | Imaging diagnosed cerebrovascular event at least 1 year after surgery, |
| **Late bleeding** | Clinically significant bleeding at least 1 year after surgery. |
| **Late reoperation** | Aortic valve reoperation (surgical or transcatheter) at least 1 year after the primary procedure. |

**Selection justification and included studies**

**Supplemental Table S2:** Excluded studies and justifications

| **Author** | **DOI or link** | **Reason for exclusion** |
| --- | --- | --- |
| Elhmidi 2014 | 10.1111/jocs.12442 | Unadjusted survival |
| Rivera 2023 | 10.1161/circ.148.suppl_1.17214 | Re-do SAVR only |
| Fialka 2023 | 10.1016/j.jcjd.2023.10.137 | Conference abstract of an already included paper |
| Fialka 2023 | 10.1016/j.cjca.2023.06.143 | Conference abstract of an already included paper |
| Guzzetti 2019 | 10.1016/j.cjca.2019.07.306 | Conference abstract of an already excluded paper |
| Johnston 2019 | 10.1161/JAHA.119.013260 | Unadjusted survival |
| Kang 2022 | 10.3390/jpm12081203 | Unadjusted survival |
| Ong 2025 | 10.3390/jcdd12010032 | Unadjusted survival/Mixes SAVR and TAVI |
| Gil Llopis 2023 | 10.3390/jcdd10020038 | Mixes SAVR and TAVI |
| Alqahtani 2017 | 10.1016/S0735-1097(17)35331-7 | Conference abstract of an already included paper |
| Hariri 2024 | 10.1016/j.jcmg.2023.06.006 | Mixes SAVR and TAVI |
| Bohbot 2018 | 10.1016/j.acvdsp.2017.11.267 | Includes untreated aortic stenosis patients |
| Bradley 2022 | 10.1213/ANE.0000000000006076 | Lack of information on the number of patients |
| Carter-Storch 2024 | 10.1016/j.jacadv.2024.100853 | Study of SAVR vs TAVI |
| Deharo 2021 | 10.1016/j.amjcard.2021.05.046 | Study of SAVR vs TAVI |
| Williams 2014 | 10.1016/j.jacc.2014.01.036 | Study of SAVR vs TAVI |
| Rubens 2023 | 10.1016/j.cjco.2022.12.001 | Unmatched survival curve |
| Guzzetti 2020 | 10.1016/j.jacc.2020.02.065 | Unadjusted survival |
| Kaier 2018 | 10.1016/j.cjca.2018.04.009 | Study of SAVR vs TAVI |
| Panichella 2024 | 10.1093/eurheartj/ehae666.3097 | Unadjusted study |
| Erinne 2021 | 10.1093/eurheartj/ehab724.1638 | Unadjusted study |
| Tribouilloy 2021 | 10.1161/JAHA.120.018816 | Includes untreated aortic stenosis patients |

**Additional Patient Data**

**Supplemental Table S3:** Baseline and procedural characteristics of individual studies

| **Study; Year** | **Age**^†^**, (F/M)** | **AR, %**  **(F/M)** | **MV, %**  **(F/M)** | **LVEF**^†^**,**  **(F/M)** | **AF, %**  **(F/M)** | **HTN, % (F/M)** | **BMI**^†^**,**  **(F/M)** | **CAD, % (F/M)** | **COPD, % (F/M)** | **DM, % (F/M)** | **CPB time**^†^**, (F/M)** | **ACC time**^†^**, (F/M)** |
| --- | --- | --- | --- | --- | --- | --- | --- | --- | --- | --- | --- | --- |
| Andrei; 2015 | 60.7/61.1 | 31/31 | 6/3 | 61/61.5 | NA | NA | 27.7/27.9 | 12.9/12.9 | NA | NA | 91.3/99.3 | 77.3/82.5 |
| Çelik; 2023 | 66.1/66.3 | 8.5/10.4 | 63.3/64.3 | NA | 12.7/12.4 | 22.4/22.2 | NA | 4.8/4.4 | 10.2/12.5 | 9.4/9.4 | NA | NA |
| Chaker; 2017 | 68/68 | 0/0 | 39.9/40 | NA | 40.8/40.5 | 64.7/64.6 | NA | 24.6/24.4 | 20.2/20.2 | 24/23.9 | NA | NA |
| Chang; 2022 | NA | NA | NA | NA | NA | NA | NA | NA | NA | NA | NA | NA |
| Duncan; 2006 | 65.6/65.6 | 19.4/17.7 | 40.1/38.4 | 57.5/57.9 | 9.5/9 | 47.8/48.3 | NA | NA | 11.6/11.6 | 13.8/13.4 | 86/88.33 | 64.3/65.3 |
| Fialka; 2024 | 67.3/66.8 | NA | 9.3/12.1 | NA | NA | 28.4/28.1 | 29.9/29.6 | 3.14/2.83 | 30/29.7 | 9.3/9.5 | 142/150.5 | 111.3/117.1 |
| Johnston; 2024 | 72.9/70.2 | 0/0 | 13.3/18.7 | NA | 15.4/16.9 | 40.6/36.3 | NA | NA | NA | 19.3/21.3 | NA | NA |
| Kulik; 2009 | 68.3/64.3 | 11/20 | 63.6/54.7 | NA | 3.9/3.3 | 58.5/50.9 | 27.7/28 | NA | NA | 5.5/4.3 | 117.1/127 | 78.1/84.9 |
| Lopez de Andres; 2019 | 72/70 | 0/0 | 48.4/48.4 | NA | 35.2/33 | NA | NA | 25.8/31.8 | 3.7/3.8 | 24.6/23.3 | NA | NA |
| Myllykangas; 2020 | 72.2/71.8 | 7.3/7.7 | 24.4/24.8 | NA | 15.1/14.7 | NA | NA | NA | NA | NA | NA | NA |
| Pawlik; 2023 | 66.6/66.3 | 18/17.4 | 59.9/59.4 | 57.7/58.3 | NA | 83.5/84.5 | 28.6/28.2 | 12.3/10.9 | 19.4/20 | 31.4/31.8 | NA | NA |
| Zierer; 2024 | 59.8/59 | 27/30 | 0/0 | 46/45 | NA | 57/56 | 27.4/27.3 | 74/72 | 9.1/8.1 | 17/17 | 102.1/103.9 | 71.7/75 |
| Hernandez; 2021 | 59.9/59.8 | 0/0 | 80.6/79.8 | 61.1/61.4 | 7.7/7.5 | 61.8/60 | 29.3/29.2 | NA | 6.4/6.4 | 21.8/22.2 | NA | NA |

**†:** mean; ***:** mean or percentage of both groups combined; **ACC:** Aortic Cross-Clamp; **AF**: Atrial Fibrillation; **AR:** Aortic Regurgitation; **BMI:** body mass index; **CAD:** coronary artery disease; **CPB:** cardiopulmonary bypass; **COPD:** chronic obstructive pulmonary disease; **DM:** diabetes mellitus; **F:** Female; **HTN:** Hypertension; **M:** Male; **MV:** Mechanical Valve; **NA:** Not Available; **LVEF:** Left Ventricular Ejection Fraction.

**Adjustments of each study**

**Supplemental Table S4:** Adjustments made in each included study.

| **Author, year** | **Adjustments made** |
| --- | --- |
| Zierer, 2024 | Comorbidities and pre-operative echocardiographic measures. |
| Pawlik, 2023 | Age and comorbidities. |
| Myllykangas, 2020 | Age, comorbidities, concomitant CABG, operation year, surgical status, and prosthesis type. |
| Lopez de Andres, 2019 | Age, comorbidities, concomitant CABG, preop pacemaker, operation year, and surgical status. |
| Kulik, 2009 | Age and comorbidities. |
| Johnston, 2024 | Age, comorbidities and coronary intervention at index valve replacement. |
| Hernandez, 2021 | Age, comorbidities and prosthesis type. |
| Fialka, 2024 | Age and comorbidities. |
| Duncan, 2006 | Age, comorbidities, prosthesis type, operation year, surgical status, prosthesis size, and operation time. |
| Chang, 2022 | Age, comorbidities and date of procedure. |
| Chaker, 2017 | Age, race and comorbidities. |
| Çelik, 2023 | Age, comorbidities, prosthesis type and size, concomitant CABG, and surgical status. |
| Andrei, 2015 | Age, comorbidities, surgeon, echocardiographic parameters, aneurysm resection, and concomitant mitral valve surgery. |

**CABG:** coronary artery bypass grafting.


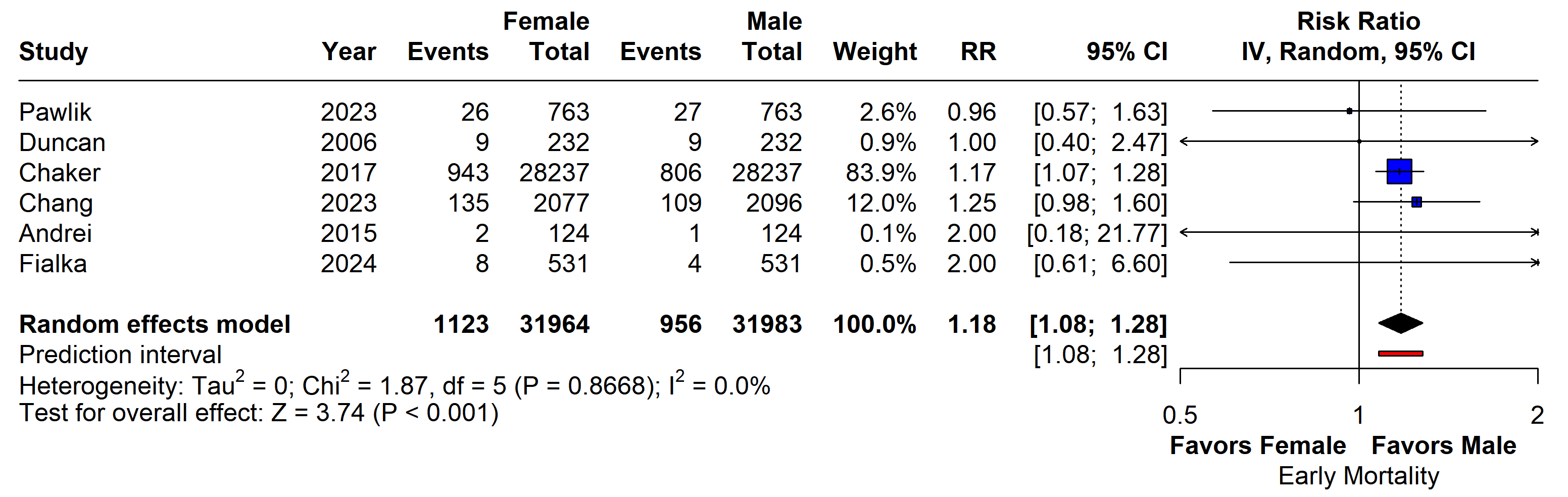


**Supplemental Figure S1**: Subgroup analysis of short-term mortality in isolated SAVR.


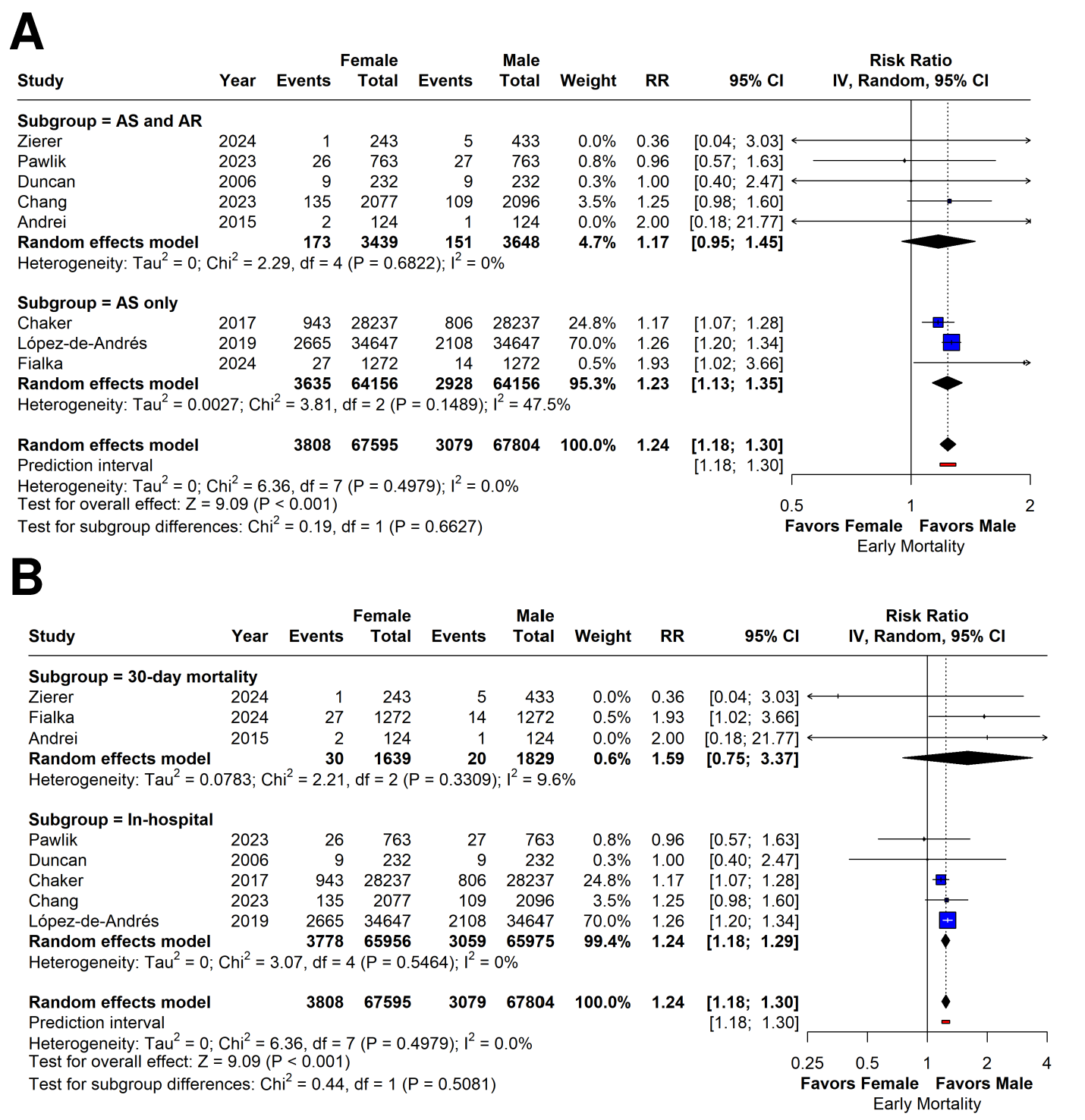


**Supplemental Figure S2**: **(A):** Subgroup analysis of short-term mortality in studies including aortic stenosis only or aortic stenosis and aortic regurgitation as indications for surgery; **(B):** Subgroup analysis of short-term mortality in studies reporting in-hospital versus 30-day mortality. **AS:** aortic stenosis; **AR:** aortic regurgitation.


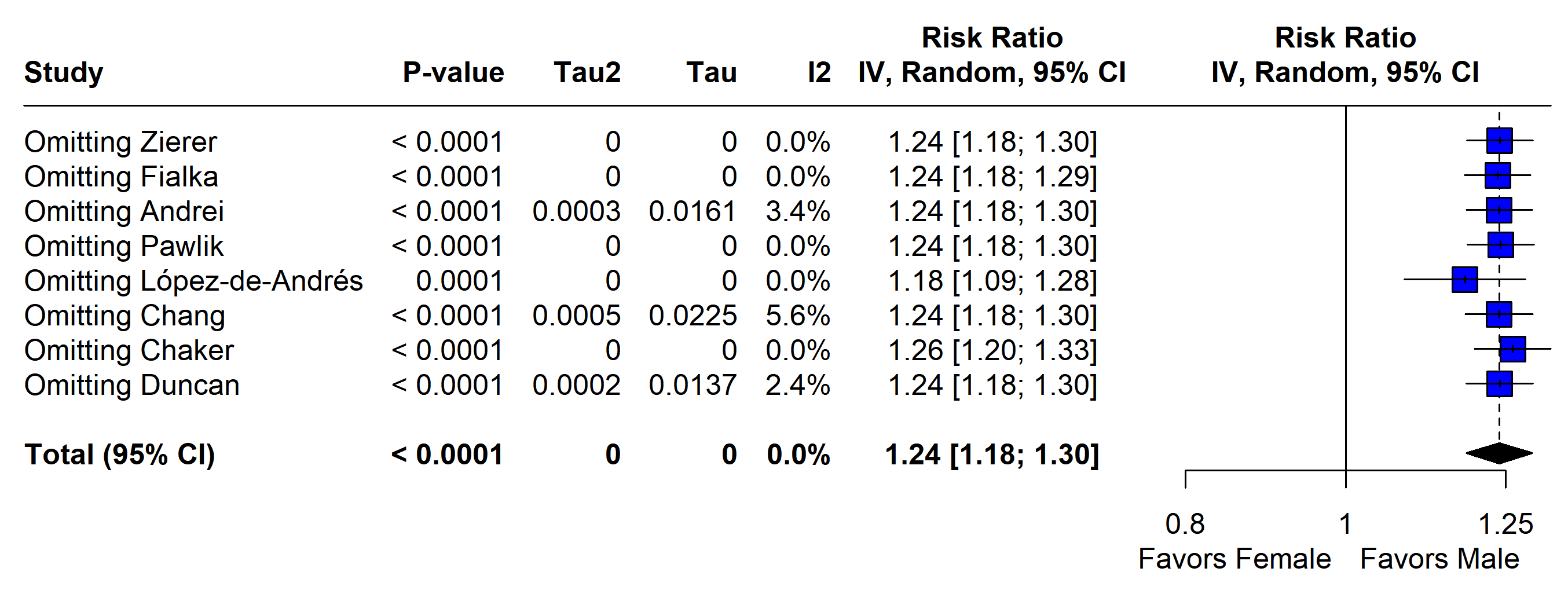


**Supplemental Figure S3:** Leave-one-out sensitivity analysis for short-term mortality.


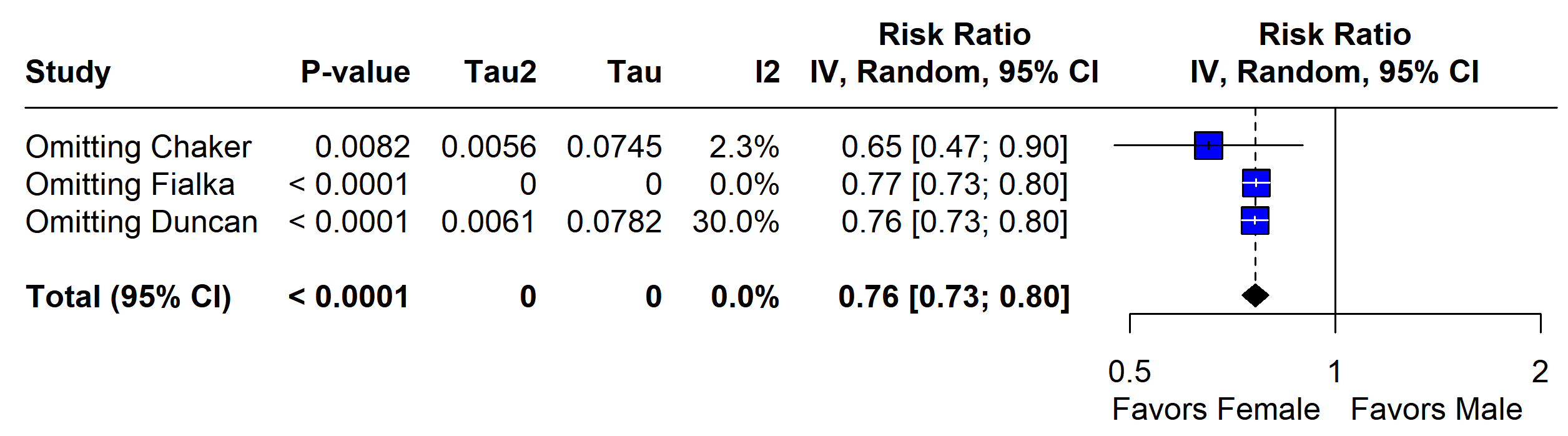


**Supplemental Figure S4:** Leave-one-out sensitivity analyses for acute kidney injury.


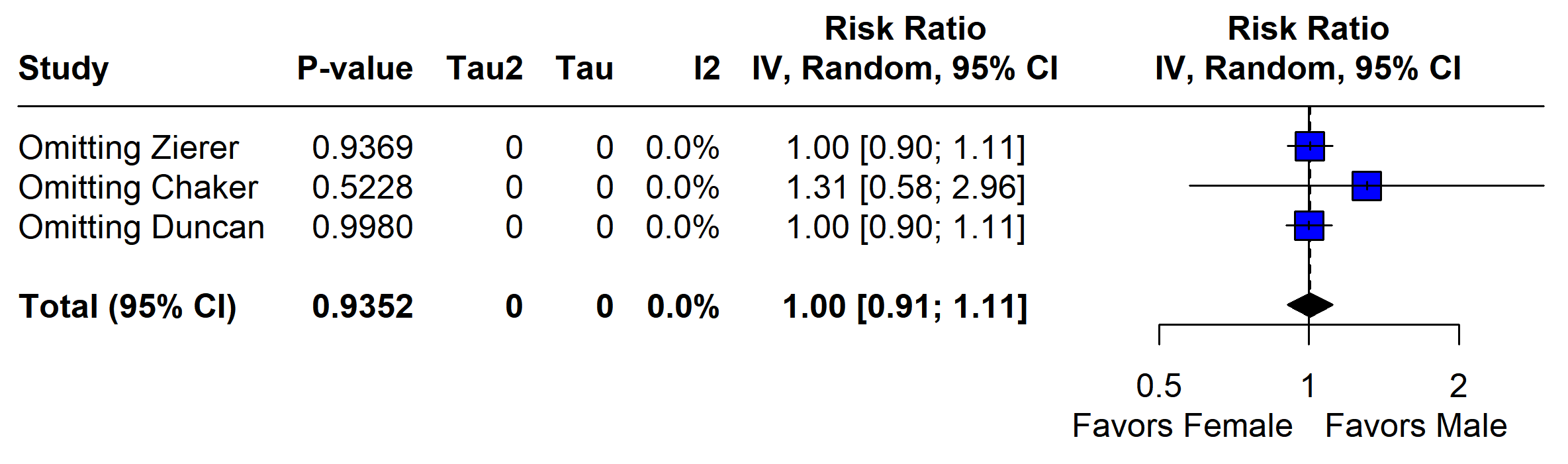


**Supplemental Figure S5:** Leave-one-out sensitivity analyses for stroke.


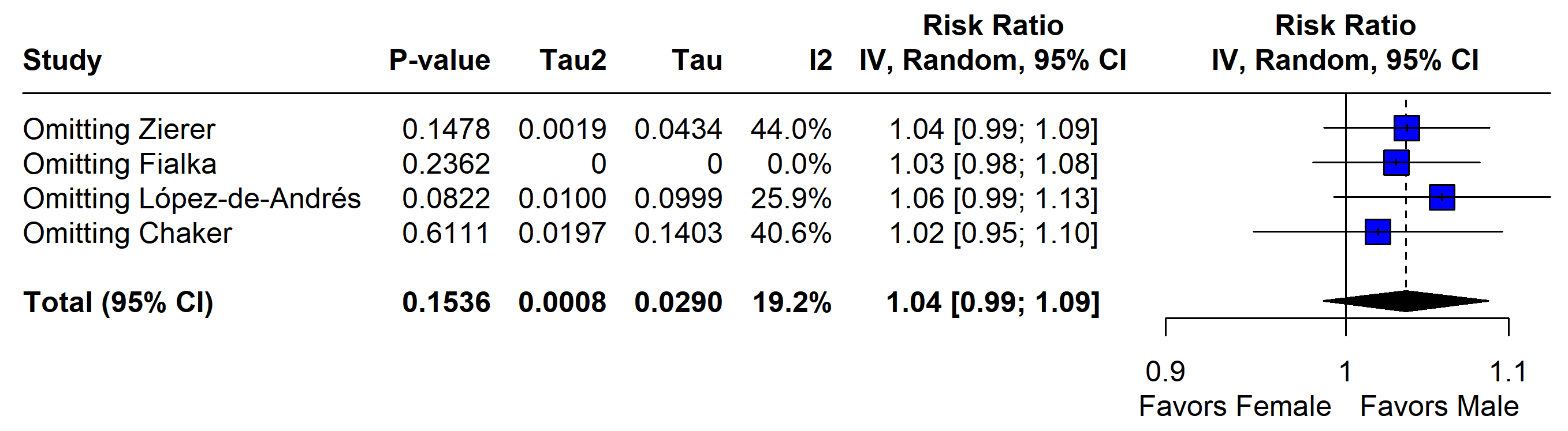


**Supplemental Figure S6:** Leave-one-out sensitivity analyses for pacemaker implantation.


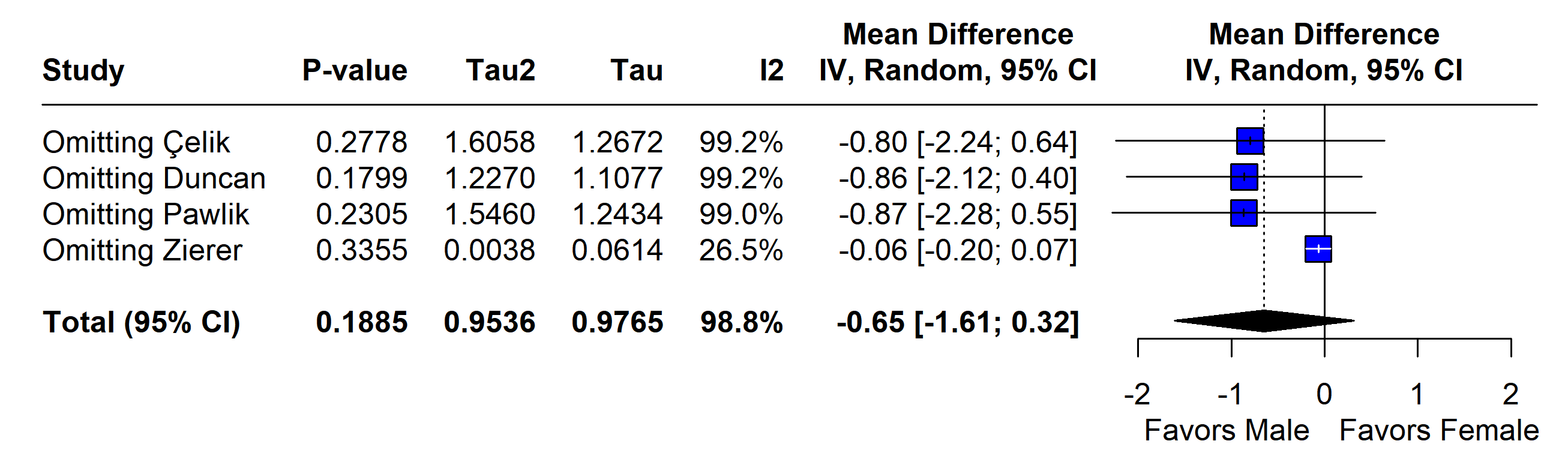


**Supplemental Figure S7:** Leave-one-out sensitivity analyses for prosthesis size.


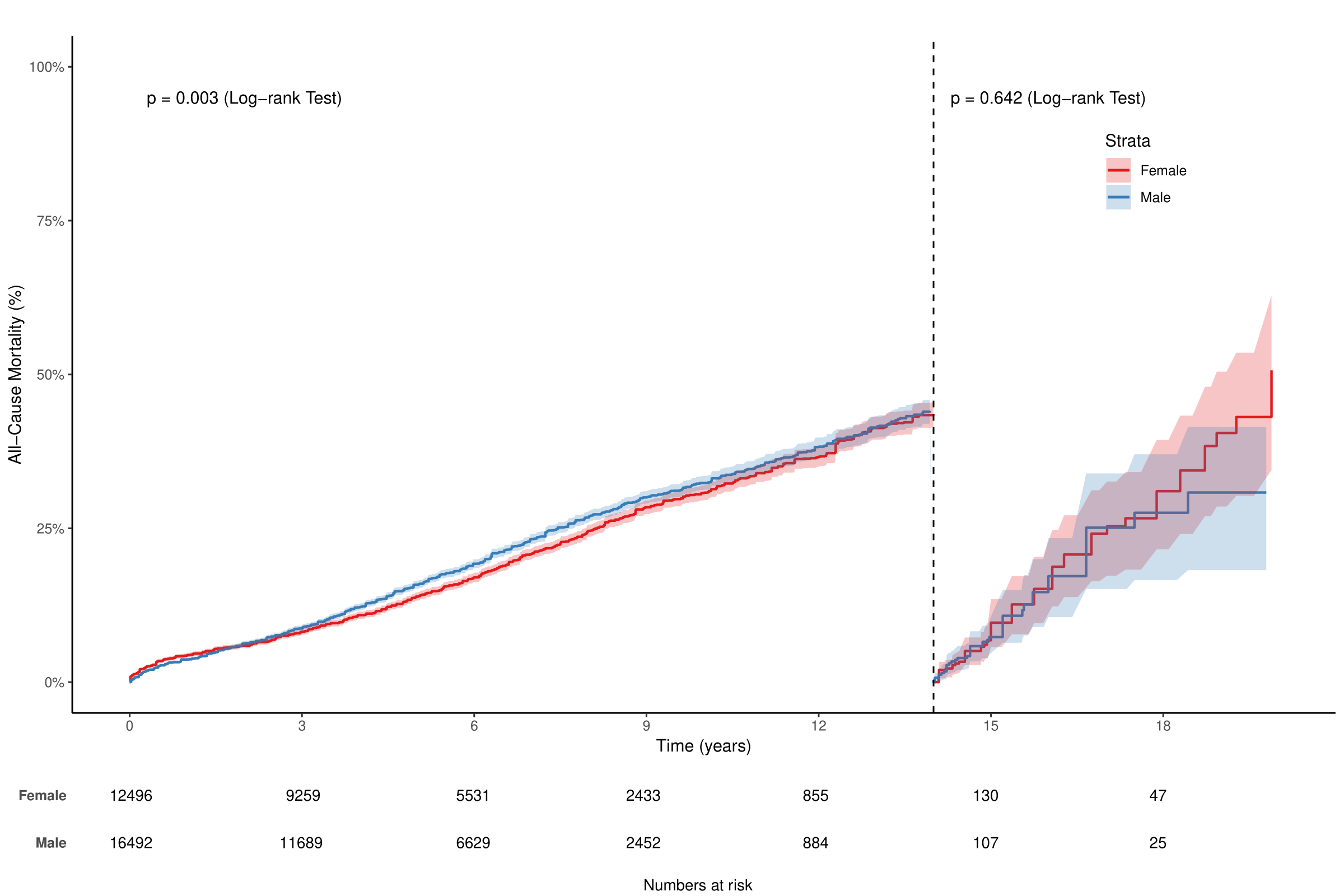


**Supplemental Figure S8:** Landmark analysis for the outcome of all-cause mortality. The curves show a survival benefit of female sex up to 14 years (p=0.003), with similar survival thereafter, until 20 years of follow-up (p=0.642). The blue line represents the female group while the red line represent males.

**
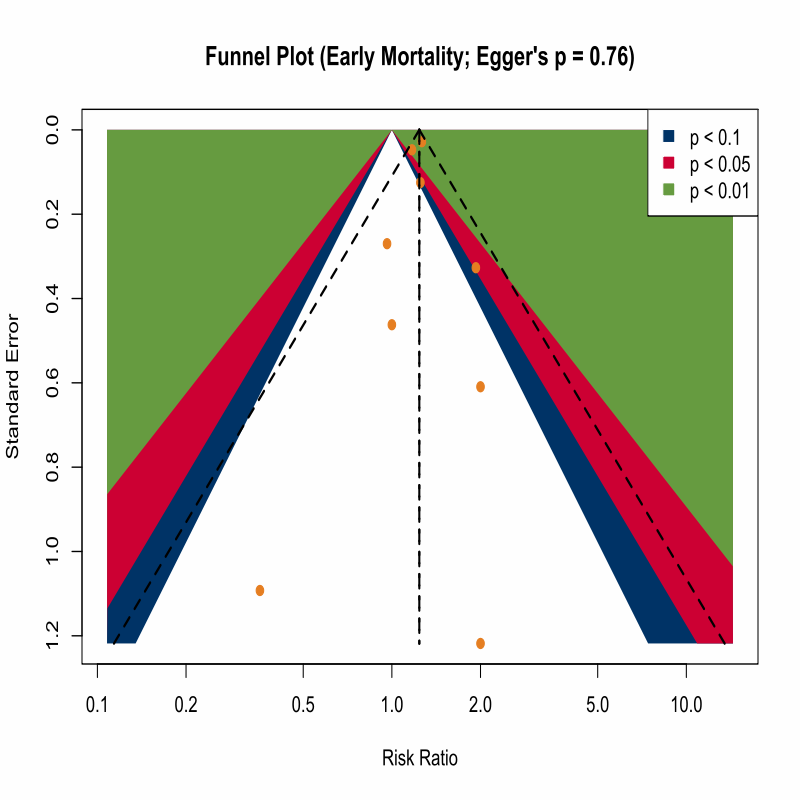
**

**Supplemental Figure S9:** Contour-enhanced funnel plot for the outcome of short-term mortality. Egger’s regression test for funnel plot asymmetry was non-significant (p=0.76).

**
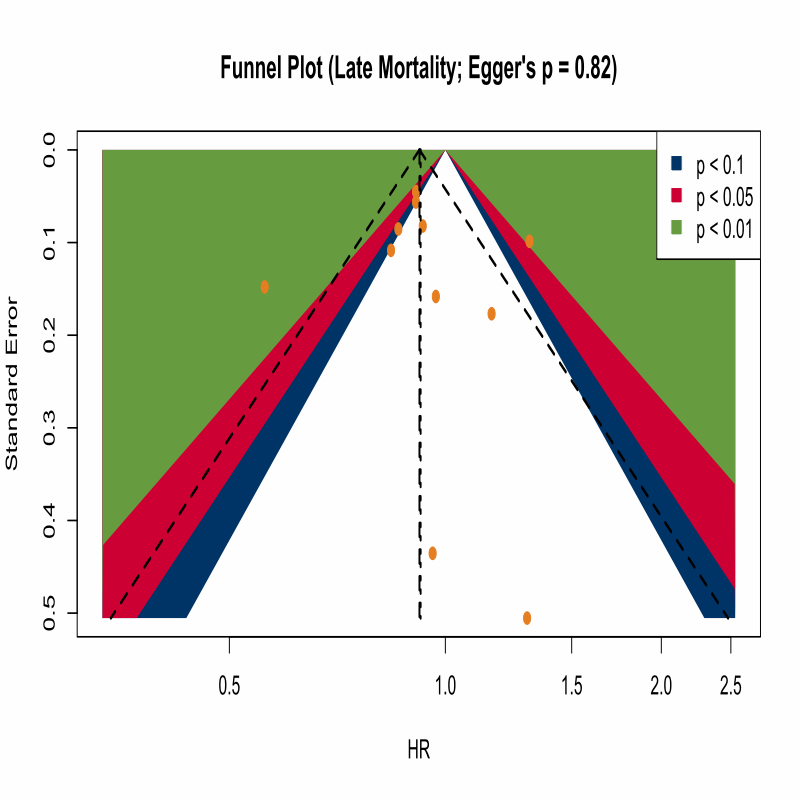
**

**Supplemental Figure S10:** Contour-enhanced funnel plot for the outcome of long-term mortality. Egger’s regression test for funnel plot asymmetry was non-significant (p=0.82).


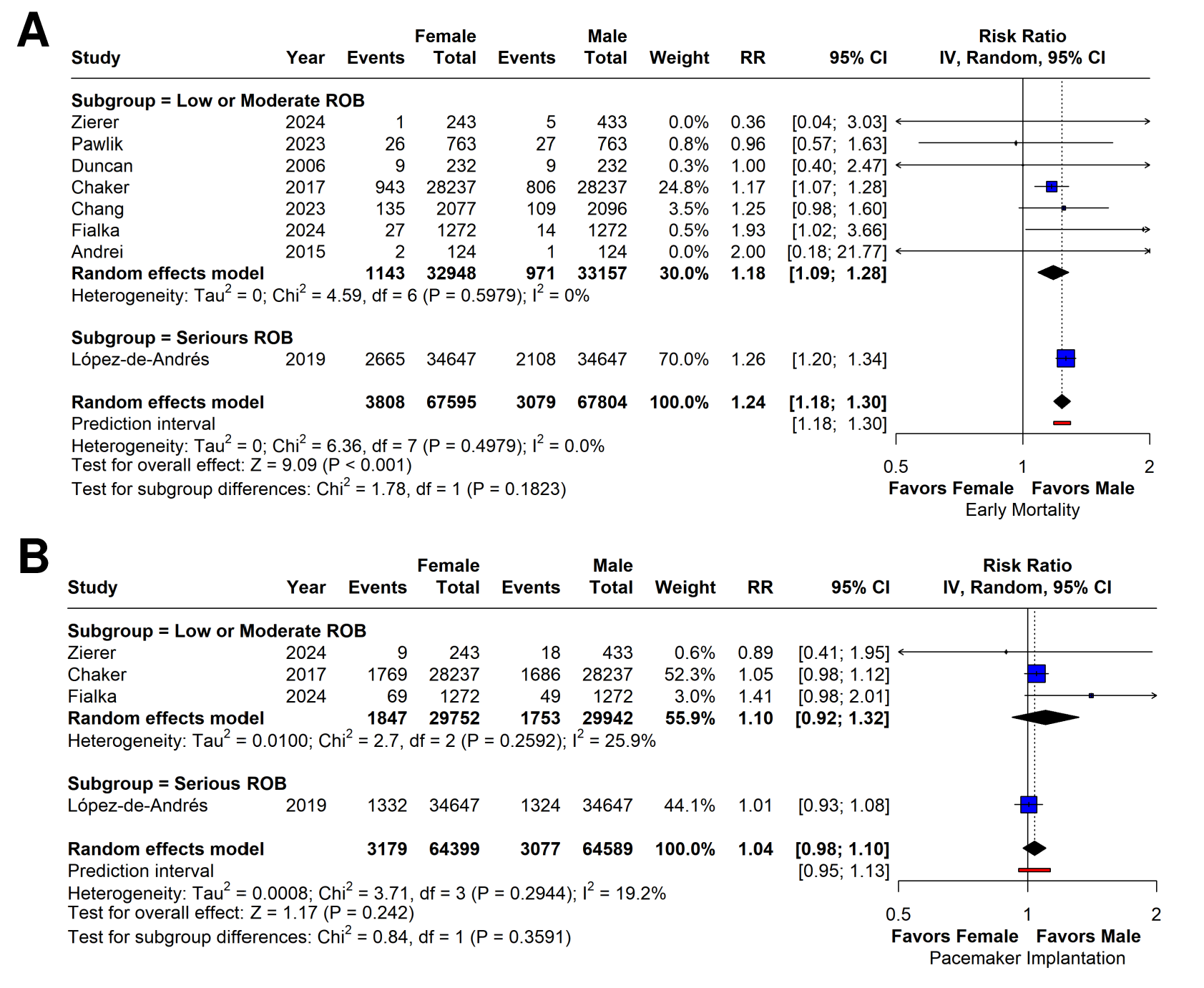


**Supplemental Figure S11: (A)** Subgroup analysis by risk of bias for the outcome of early mortality**; (B)** Subgroup analysis by risk of bias for the outcome of permanent pacemaker implantation.

**
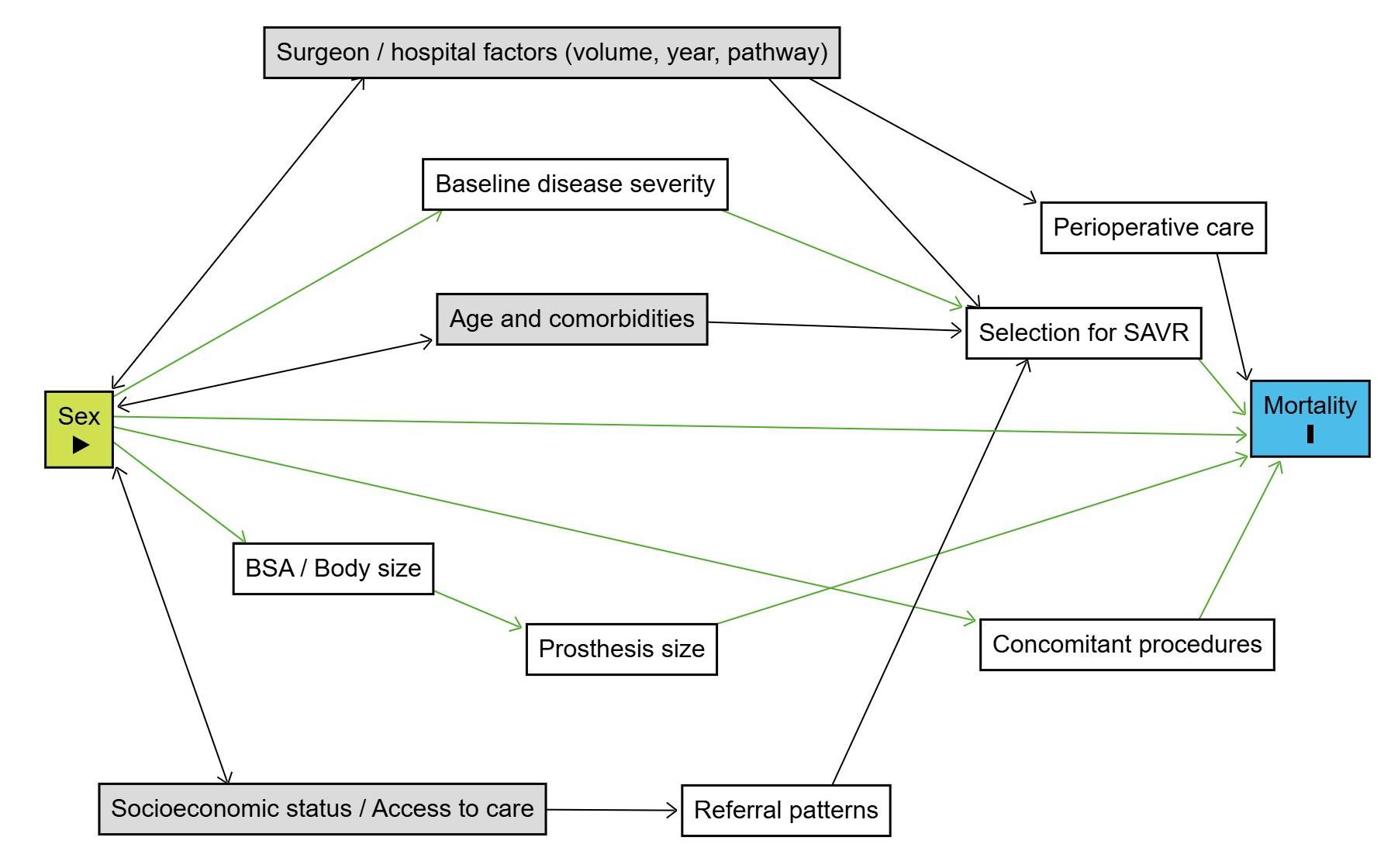
**

**Supplemental Figure S12:** Direct acyclic graph for the association between the exposure (patient sex) and the main outcome (mortality). Green lines point to potentially causal relations, while black lines point to confounding relations. Boxes in gray are confounders that can be adjusted to reduce bias. Boxes in white represent mediators and colliders that should not be adjusted for. **BSA:** body surface area; **SAVR:** surgical aortic valve replacement.

**
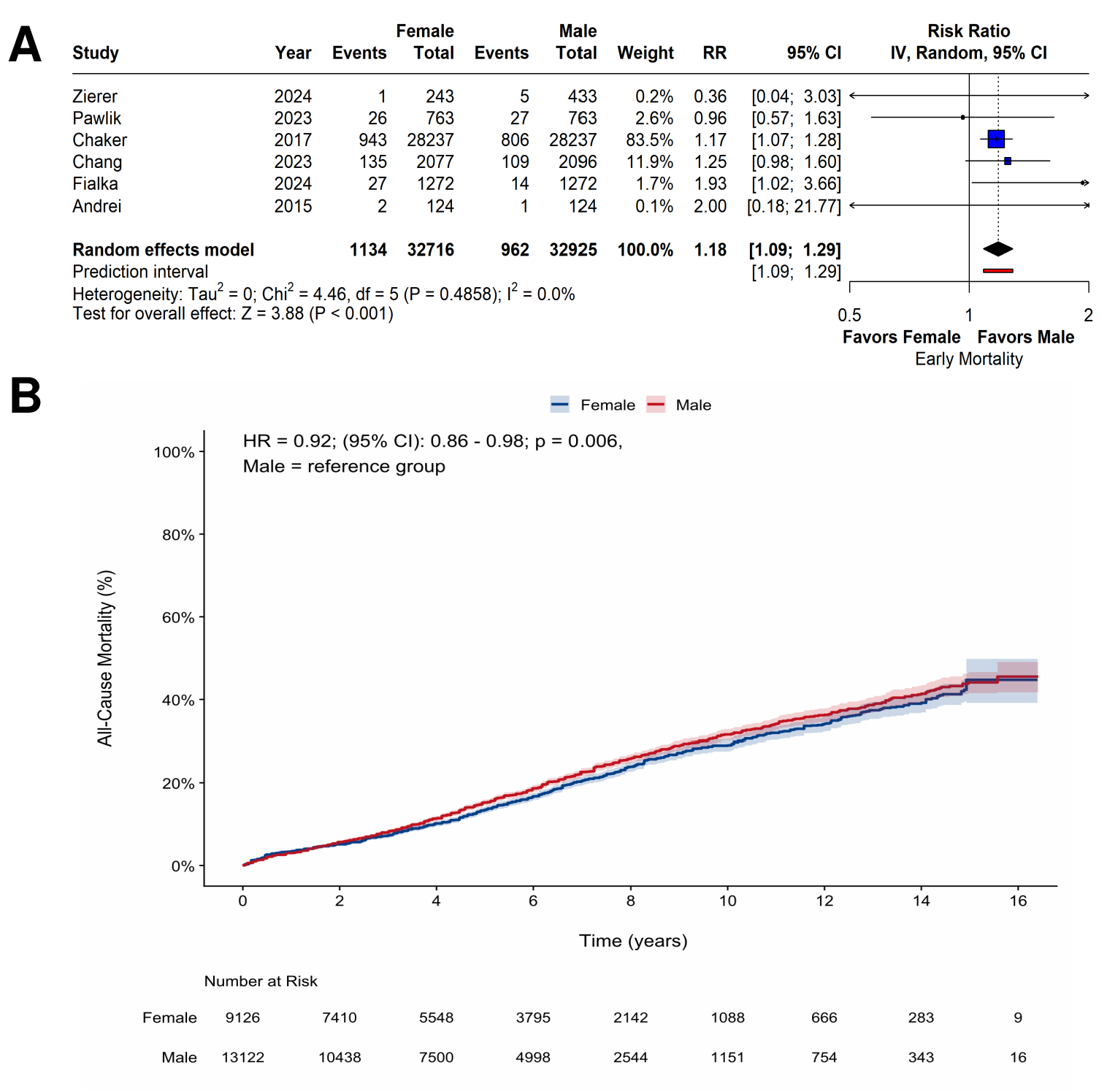
**

**Supplemental Figure S13:** Subgroup analysis of early (A) and late (B) mortality following SAVR in studies that investigate the total effect of sex on outcomes of SAVR. Similarly to the main analysis, female sex was associated with higher risk of early mortality, but lower hazards of late mortality.
